# Supplementary material for: Genetic and phylogenetic uncoupling of structure and function in human transmodal cortex
Source: Nat Commun. 2022 May 9;13:2341. doi: 10.1038/s41467-022-29886-1 (PMC9085871; doi:10.1038/s41467-022-29886-1)
Supplement: Supplementary file 1 — Supplementary Information [file 41467_2022_29886_MOESM1_ESM.pdf]

GENETIC AND PHYLOGENETIC UNCOUPLING OF STRUCTURE AND FUNCTION IN HUMAN TRANSMODAL CORTEX

SUPPLEMENTARY INFORMATION

Sofie L. Valk[1,2,3], Ting Xu[4], Casey Paquola[5,6], Bo-yong Park[5,7], Richard A.I. Bethlehem[8], Reinder Vos de Wael[5], Jessica Royer[5], Shahrzad Kharabian Masouleh[2,3], Şeyma Bayrak[1], Peter Kochunov[9], B.T. Thomas Yeo[10-14], Daniel Margulies[15], Jonathan Smallwood[16], Simon B. Eickhoff[2,15*], Boris C. Bernhardt[5*]

1. Otto Hahn Group Cognitive Neurogenetics, Max Planck Institute for Human Cognitive and Brain Sciences, Leipzig, Germany; 2. INM-7, FZ Jülich, Jülich, Germany; 3 Institute of Systems Neuroscience, HHU Duesseldorf, Duesseldorf, Germany;4. Center for the Developing Brain, New York City, USA; 5. Multimodal Imaging and Connectome Analysis Lab, McConnell Brain Imaging Centre, Montreal Neurological Institute and Hospital, McGill University, Montreal, Quebec, Canada; 6. INM-1, FZ Jülich, Jülich, Germany; 7. Department of Data Science, Inha University, Incheon, South Korea; 8. Department of Psychiatry, Cambridge University, Cambridge UK; 9. University of Baltimore, Baltimore, USA; 10. Department of Electrical and Computer Engineering, National University of Singapore, Singapore, Singapore; 11. Centre for Sleep and Cognition (CSC) & Centre for Translational Magnetic Resonance Research (TMR), National University of Singapore, Singapore, Singapore; 12. N.1 Institute for Health & Institute for Digital Medicine (WisDM), National University of Singapore, Singapore, Singapore; 13.Martinos Center for Biomedical Imaging, Massachusetts General Hospital, Charlestown, Massachusetts, United States of America; 14 Integrative Sciences and Engineering Programme (ISEP), National University of Singapore, Singapore, Singapore; 15 Neuroanatomy and Connectivity Lab, Institut de Cerveau et de la Moelle epiniere, Paris, France; 16 Department of Psychology, Queen's University, Kingston, Ontario, Canada

Correspondence to
Sofie L Valk, PhD
e. s.valk@fz-juelich.de
* These authors contributed equally

SUPPLEMENTARY NOTE 1

Replication and robustness analysis (Supplementary Fig 2 and 3)

We performed replication analysis in a second sample having both rsfMRI and T1q available in 50 healthy individuals (21 women; age mean±SD=29.82±5.73 years) (MICS: https://portal.conp.ca/dataset?id=projects/mica-mics). Overall, we found highly convergent patterns (coupling MPC-FC: r=0.736, MPCG1-rsG1: r=0.615). Notably, we observed a difference within the paralimbic network, with the 'ventral attention' network being more differentiated from the 'limbic' network as defined in [1]. Also, when analyzing the HCP data using the Glasser parcellation scheme we observed similar dissociations between heteromodal and paralimbic regions as observed using the Schaefer 400 parcellation.

Heritability and variance of principle gradient of MPC and rsFC (Fig 5)

We evaluated whether heritability of rsFC and MPC showed similar patterning as gradients over the mean of each respective measure. Overall, we found highly similar patterns. Principal axes of mean and heritable highly correlated for both MPC (r=0.808, p_{spin}<0.0001) and for rsFC (r=0.892, p_{spin}<0.0001) and regions at similar levels of the gradient showed also heightened heritability of MPC/rsFC with regions places at similar gradient levels. Then we studied whether also the variance (standard deviation) of both measures followed a similar organizational pattern. Indeed, also here we found a close association between the mean gradient pattern and its variance (MPC = 0.98, rsFC = 0.78), indicating that also the individual variation varied along these organizational patterns.

Macaque gradient validation (Supplementary Fig 4)

The principal gradient of MPC in macaques showed a sensory-fugal axis in organization of T1wT2w profiles with an apex in superior frontal and sensory-motor regions on the one hand, and an apex in posterior cingulate and inferior temporal lobe on the other hand. The principal gradient of macaques and its human correspondence had a positive correlation (Spearman's r=0.268, p <0.005). Follow-up analysis indicated this gradient correlated positive with profile skew (Spearman's r=0.852, p<0.001), rather than the mean regional T1wT2 (Spearman's r=0.111, p>0.05) (**Supplementary Fig 4**), similar to previous work [2]. Second, based on previous work (Xu, in prep) we selected the third gradient of rsFC in macaque monkeys to reflect the principal gradient of rsFC in humans. In the current approach, based on averages in the Markov parcellation [3], we also observed a positive correlation between the aligned human

and macaque gradients (Spearman's r=0.313, p<0.001, unaligned human FC gradient: Spearman's r 0.14, p=0.0571).

Robustness evaluations of human- macaque differences

Overall, tSNR varied between species, with macaques showing higher tSNR than humans. When mapping human tSNR to macaque space [4] and computing the difference per region, we also found widespread increases in tSNR in macaques compared to humans across all regions, in particular in visual cortex. However, correlating tSNR between species, we observed no spatial relationship (Spearman's r=0.03) nor a relationship between difference in tSNR and difference in coupling between species (Spearman's r=0.00). This suggests that the observed uncoupling of MPC and rsFC in humans cannot be accounted for by variations in tSNR alone (**Supplementary Fig. 5**).

To evaluate whether the observed structure-function coupling in macaques might be due to awake versus anesthetized status, as well as differences in age-range and sex between samples, we evaluated two additional macaque samples, the Newcastle and Oxford sample (**Fig. 4**). As these datasets did not have high-resolution microstructural MRI data available, we correlated the region-wise average functional connectivity data of the two additional samples, thought to be most impacted by anesthesia, with the microstructural profile covariance data of the Davis sample. Despite some inter-site differences, patterns of decoupling were similar for anesthetized and awake samples with different sex and age distributions (Davis-Newcastle: r=0.78; Davis-Oxford: r=0.90; Newcastle-Oxford: r=0.71). All samples showed correlation with the human HCP measure of structure-function coupling (Davis: Spearman's r=0.48, p_{spin}=0.012; Newcastle: Spearman's r=0.36, p_{spin}=0.06; Oxford: Spearman's r=0.40, p_{spin}=0.05). For all macaque samples, we observed similarly high coupling in idiotypic, unimodal and heteromodal regions, whereas only paralimbic regions were uncoupled. Conversely, in human data we observed relative uncoupling in unimodal, heteromodal and paralimbic regions (**Supplementary Table 1, Table 2**).

Computing robustness of gradients as a function of varying alpha [0 1] and diffusion time [0 9] we found gradients to be highly similar (r>0.99) to each other in human and macaque data (**Supplementary Fig. 6**).

Individual variation in humans

Probing inter-individual variation of microstructure-function coupling in humans, we observed a high correspondence between single-subject and group-average findings (mean r:

0.72 range: [0.38 0.86]), indicating consistency. Also evaluating potential effects of ICV on structure-function coupling, we observed positive and negative associations between coupling and ICV (stronger coupling in left superior frontal and right posterior insular regions (FDRq<0.05) and decreased coupling in right posterior temporal regions (FDRq<0.05)). Importantly, however, there was only a weak and non-significant link with mean patterns of structure-function coupling (r=0.12, p_{spin}>0.1). Combined, these analyses suggest that our results are robust at the level of the individual and against variations in ICV (**Supplementary Fig 8**).

To study whether observed patterns of structure-function coupling in humans were similar between males and females we evaluate coupling patterns in both sexes (**Supplementary Fig 9**). We observed highly similar patterns of coupling and uncoupling (r=0.99). At the same time, we found also differences as function of sex at the individual level, notably increased coupling in superior frontal and parietal regions and decreased coupling in temporal, cingulate and medial frontal regions in males relative to females (FDRq<0.05).

To evaluate whether coupling and gradient differences in structure and function were meaningful at the individual level for behavior, we correlated relevant behavioral scores of the HCP dataset with individual maps of coupling and MPC-rsFC G1 difference and assessed whether t-values correlated with the coupling and gradient difference pattern. We found that self-efficiency, friendship and extraversion related to strong uncoupling, e.g. more coupling in sensory regions and uncoupling in transmodal regions. Along the axes dissociating heteromodal from paralimbic transmodal regions, we found a higher similarity (decreased differences) of MPC G1 and rsFC G1 could be associated with stress, sadness, pain and neuroticism. In turn, increased difference between both gradients was related to working memory, cognition and card sorting. These patterns should be interpreted differently from the mapping with meta-analytical task activations. However, they are again suggestive of meaningful dimensions in structure-function coupling and transmodal uncoupling related to higher-order human cognition (**Supplementary Fig 10**).

To evaluate the potential impact of ethnicity on heritability, we reran the heritability analysis in individuals whose self-reported race was 'White' (n=749 individuals in our main sample). Indeed, similar patterns of 'genetic uncoupling' were observed as in the complete sample rsFC-$rsFC_{heritability}$: r=0.95 and MPC-$MPC_{heritability}$: r=0.93, $rsFC_{G1}$ r: 0.975 and MPC_{G1} r:0.935 (**Supplementary Fig 11**).

Alternative heritability computations considering intra-individual variation (Supplementary Fig 12)

Computing heritability using an alternative measure suggested to account for intra-individual variation [5], we observed overall consistent patterns. Specifically, we observed strong heritable coupling in idiotypic (Spearman's r=0.789±0.121) and unimodal (Spearman's r=0.704±0.162) regions, whereas coupling in heteromodal (Spearman's r=0.580±0.175) and paralimbic (Spearman's r=0.433±0.245) regions was reduced. These patterns correlated with MPC-rsFC coupling (Spearman's r=0.416, p_{spin}=0.01) and Solar-based heritability metrics (Spearman's r=0.754, p<0.001).

Reliability using test-retest analysis

Evaluating test-retest reliability of MPC, rsFC and coupling of MPC and rsFC we observed overall moderate test-retest reliability across all measures (0.430±0.059; rsFC ICC: 0.549±0.077; coupling: 0.568±0.221). At the same time, we found no relationship (Spearman's r=-0.02) between coupling of MPC and rsFC and test-retest reliability, suggesting uncoupling of both measures does reflect reduced reliability of both (**Supplementary Fig. 13**).

To control for the possibility that the association between mean and heritability of the measurements was related to an association with noise, measured by means of test-retest reliability in the retest sample of the HCP dataset, going in the same direction, we computed the parcel-wise relationship between each measure and their respective reliability, as quantified by ICC. Here we observed that while there was variation across parcels in the relationship between mean and reliability for rsFC (mean±SD: 0.26±0.15), this pattern was not correlated with the association between mean and heritable FC (Spearman's r=0.04, p>0.1). Performing the same analysis of MPC, we found correspondence between mean MPC and the ICC of MPC (mean±SD: 0.66±0.09), indicating that regions with similar microstructural profiles overall showed high test-retest reliability. Again, we found no relationship between mean MPC – reliability of MPC and mean MPC-heritability of MPC (Spearman's =0.18, p_{spin}>0.1).

To assess whether both gradients and their difference also reflected variation in noise, we evaluated the test-retest reliability of each gradient and their difference using ICC (**Supplementary Fig 14**). Overall, gradients were reliable (MPC_{G1} ICC mean±std: 0.71±0.21; $rsFC_{G1}$: 0.60±0.23; MPC_{G1} – $rsFC_{G1}$: 0.66±0.20) and we observed no relationship between reliability and gradient loadings (MPC_{G1} r= 0.20, p_{spin}>0.1; $rsFC_{G1}$ r=0.19, p_{spin}>0.1; MPC_{G1} – $rsFC_{G1}$ r=0.00).

SUPPLEMENTARY FIGS

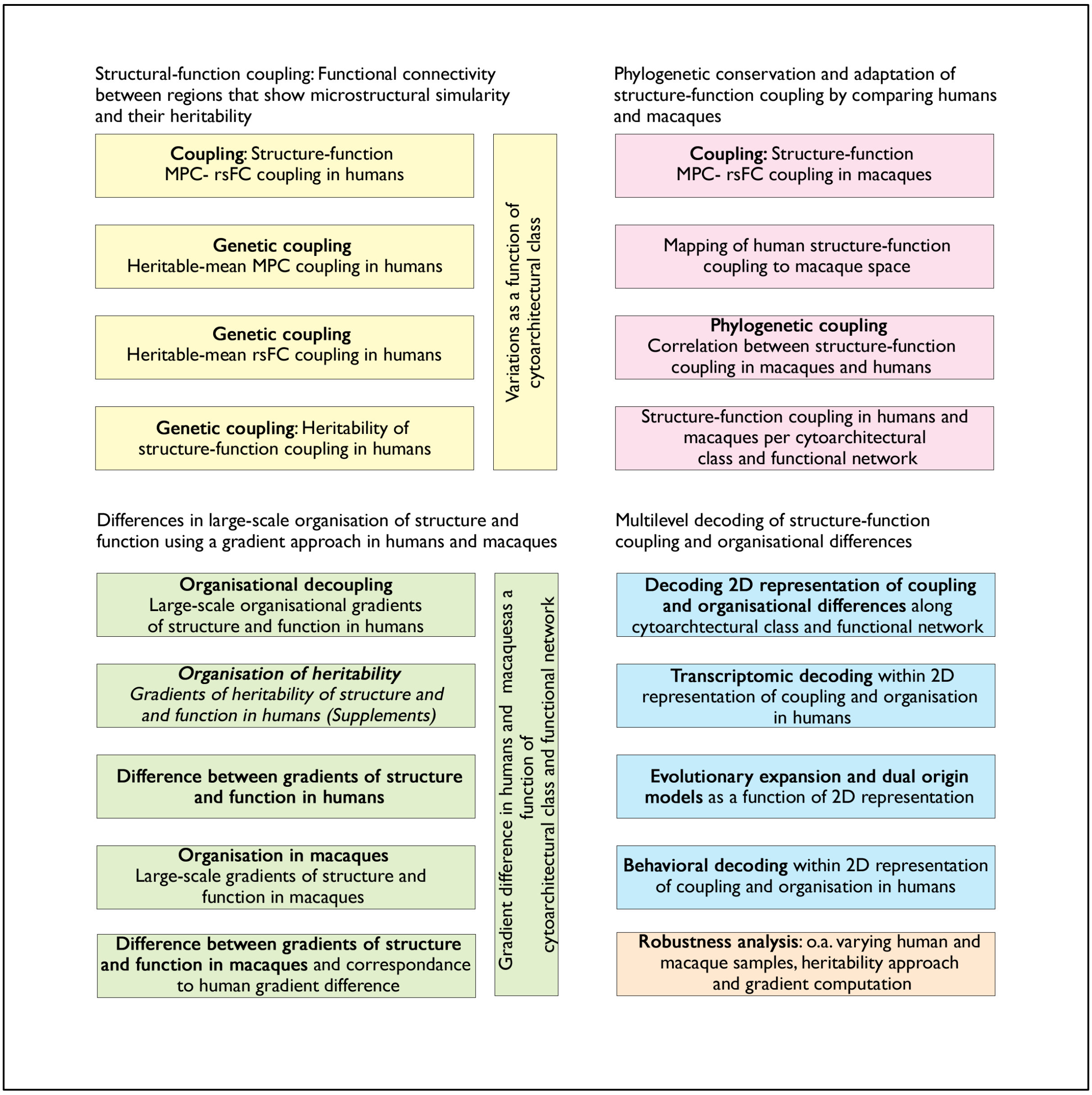

Supplementary Fig 1. Analysis scheme. Analysis scheme colored by question and analysis type.

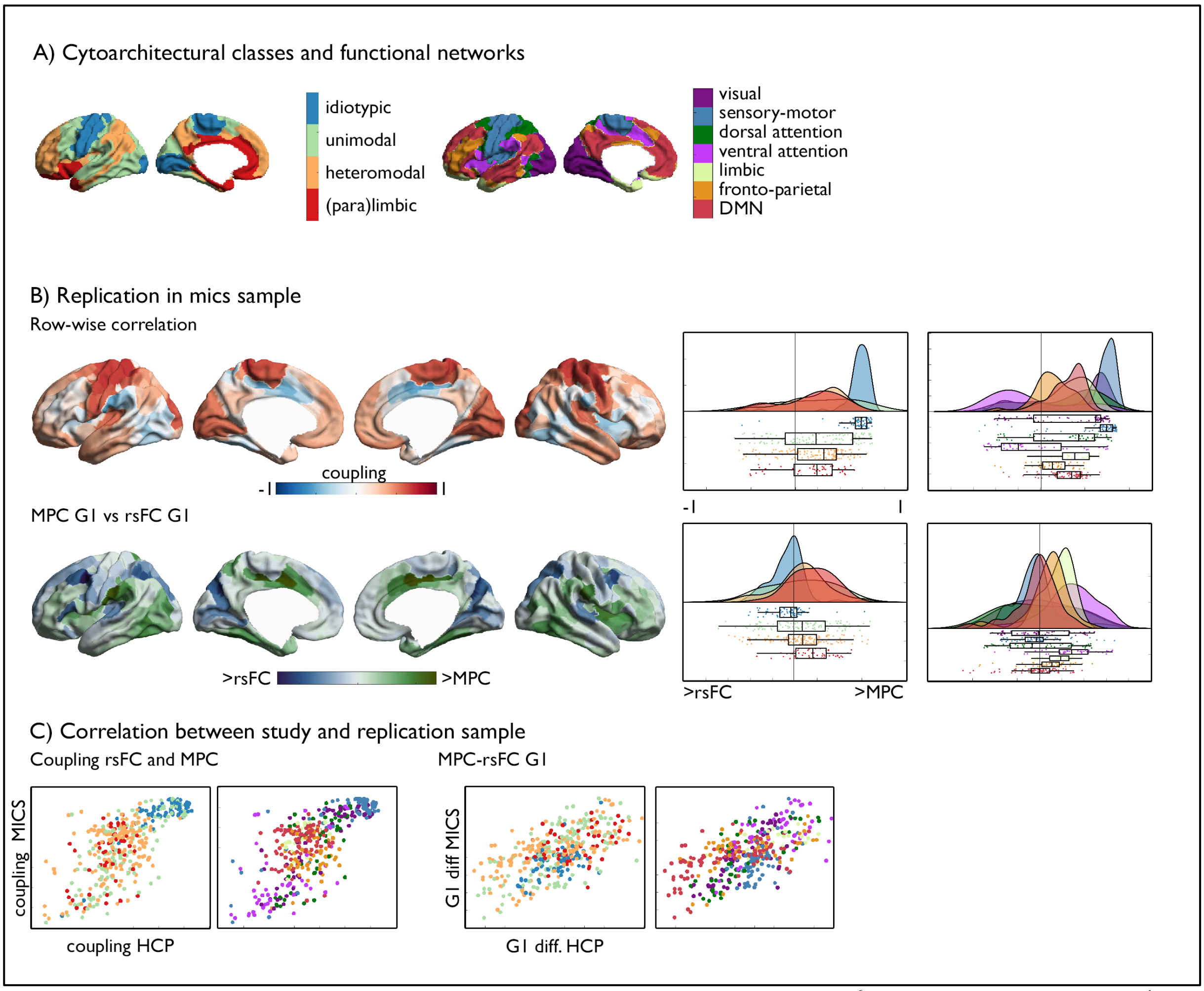

Supplementary Fig 2. Replication sample MICS. A) Cytoarchitectural class [6], and functional networks [1]; **B**) Replication in the mics sample; *upper:* row-wise correlation and associated raincloud plots of 400 parcels in cytoarchitectural class (left) and functional networks (right), boxes show the median and interquartile (25-75%) range, whiskers depict the 1.5*IQR from the quartile; *lower:* difference between gradients and associated raincloud plots in cytoarchitectural class (left) and functional networks (right); **C**) Correlation between HCP 1200 and mics sample of MPC-rsFC edge-level correlation (left) and MPC-rsFC principal gradient difference (right), colored by cytoarchitectural class and functional network. Source data are provided as a Source Data file.

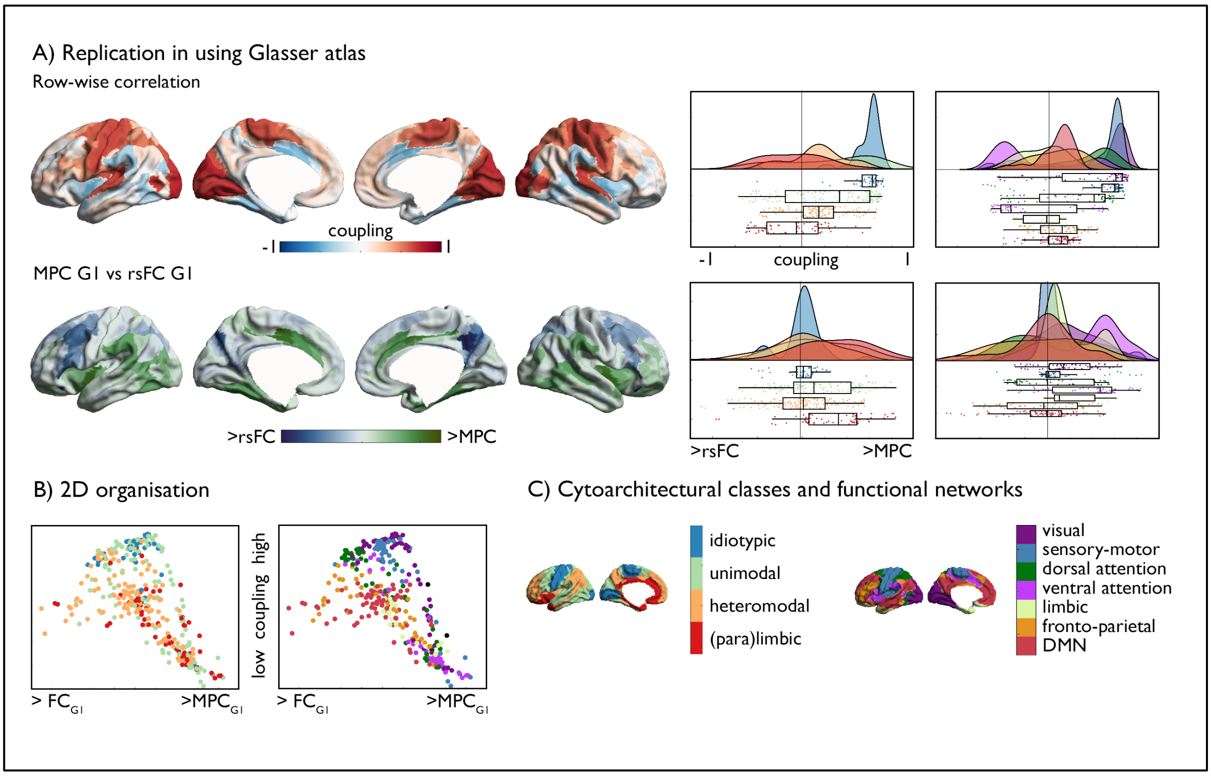

Supplementary Fig 3. Replication in HCP using the Glasser atlas. A) Replication using the Glasser parcellation; *upper:* row-wise correlation and associated raincloud plots of 400 parcels in cytoarchitectural class (left) and functional networks (right), boxes show the median and interquartile (25-75%) range, whiskers depict the 1.5*IQR from the quartile; *lower:* difference between gradients and associated raincloud plots in cytoarchitectural class (left) and functional networks (right); **B**) 2D organization x-axis gradient difference and y-axis structure-function row-wise correlation, colored by cytoarchitectural class (left) and functional network (right); **C**) Maps of cytoarchitectural class [6], and functional networks [1]. Source data are provided as a Source Data file.

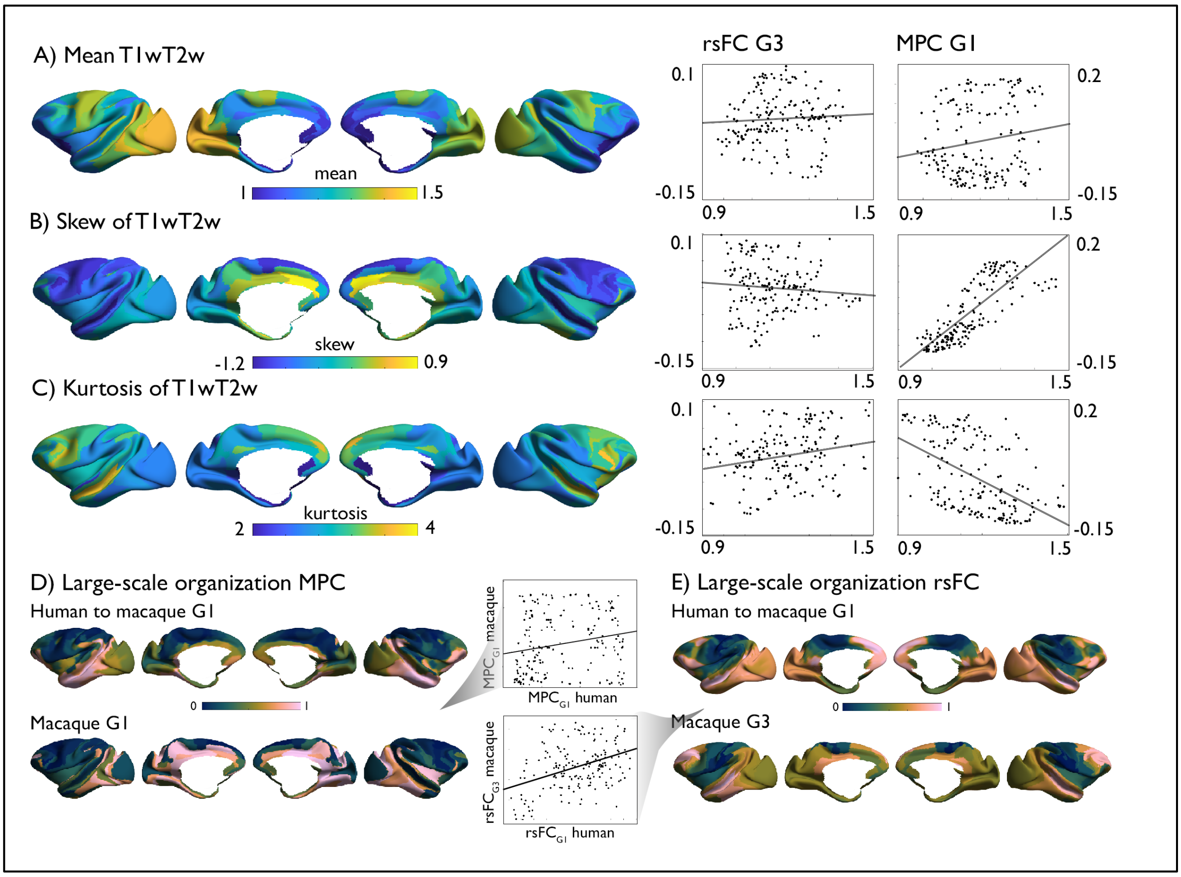

Supplementary Fig 4. Macaque gradient validation. Validation of macaque MPC and rsFC gradients, using **A-C**) mean T1wT2w, skew, and kurtosis; **D**) Large-scale organization of MPC in human-to-macaque (upper) and macaque (lower) and its correlation; **E**) Large-scale organization of rsFC in aligned-human-to-macaque (upper) and macaque (lower) and its correlation. Source data are provided as a Source Data file.

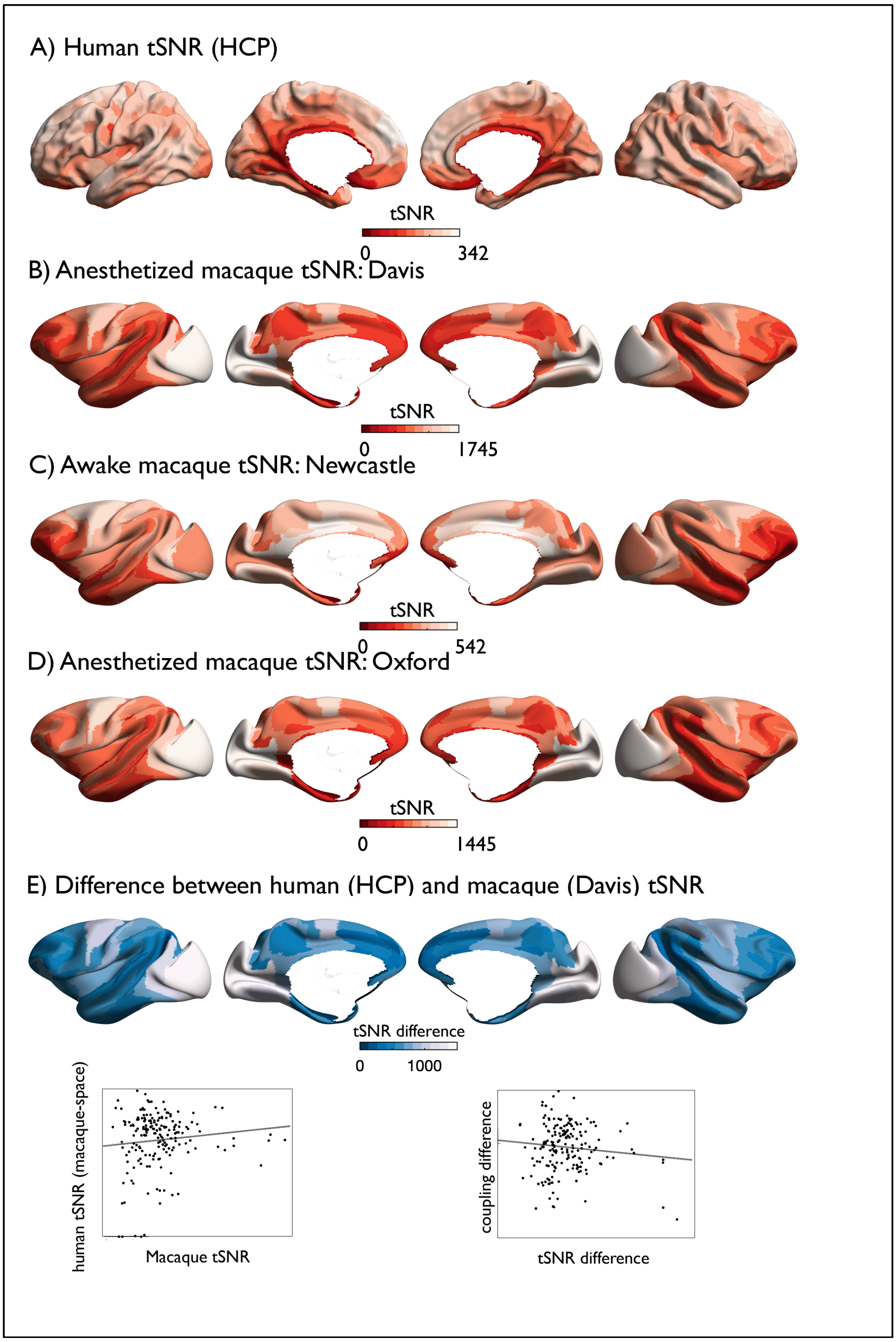

Supplementary Fig 5. tSNR in humans and macaque samples. A-D). Cortical tSNR (mean./SD) in the main human (HCP) and macaque sample (Davis, anesthetized) as well as two additional macaque samples (Newcastle, awake; Oxford, anesthetized); **E)** Difference in tSNR between humans mapped to macaque space (HCP) and macaques (Davis, main analysis sample), their correlation (left lower scatterplot), and correlation of difference in tSNR and structure-function coupling differences (right lower scatterplot). Source data are provided as a Source Data file.

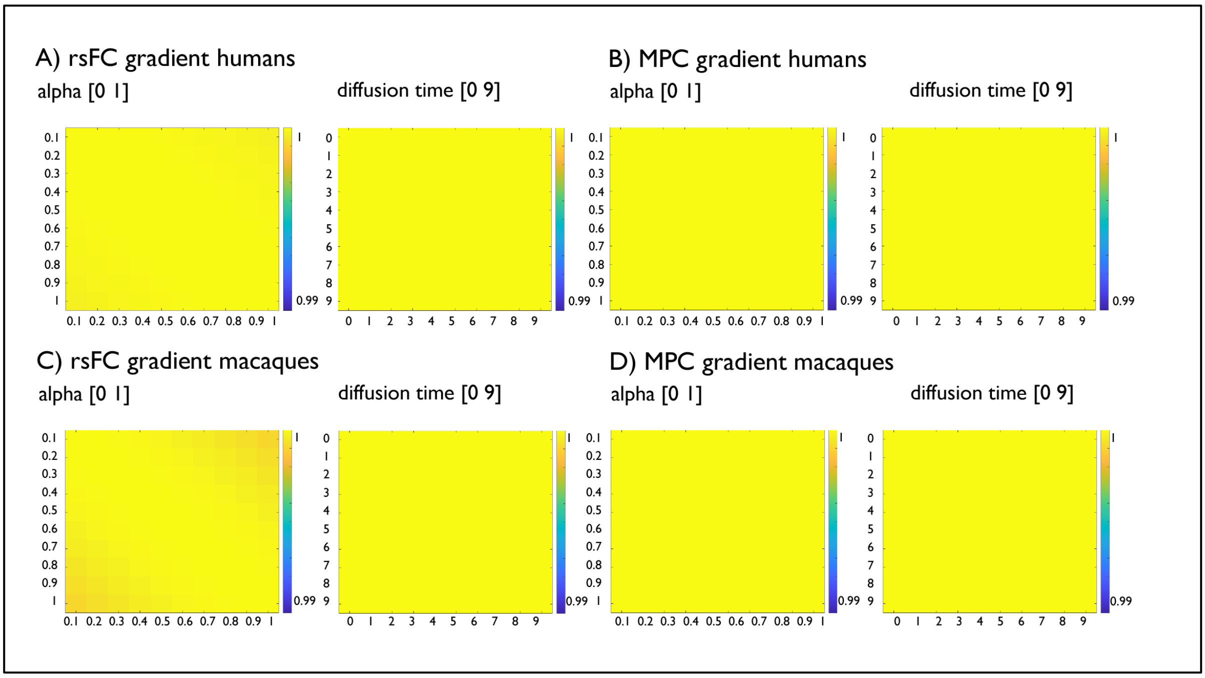

Supplementary Fig 6. Intercorrelation of gradients as a function of varying alpha and diffusion-time. A) Varying alpha and diffusion time for rsFC gradient in humans; **B)** Varying alpha and diffusion time for MPC gradient in humans; **C)** Varying alpha and diffusion time for rsFC gradient in macaques; and **D)** Varying alpha and diffusion time for MPC gradient in macaques. The parameters used for main analyses in humans and macaques in the current study are 0.5 alpha and 0 diffusion time, similar to previous work [7,8]. Source data are provided as a Source Data file.

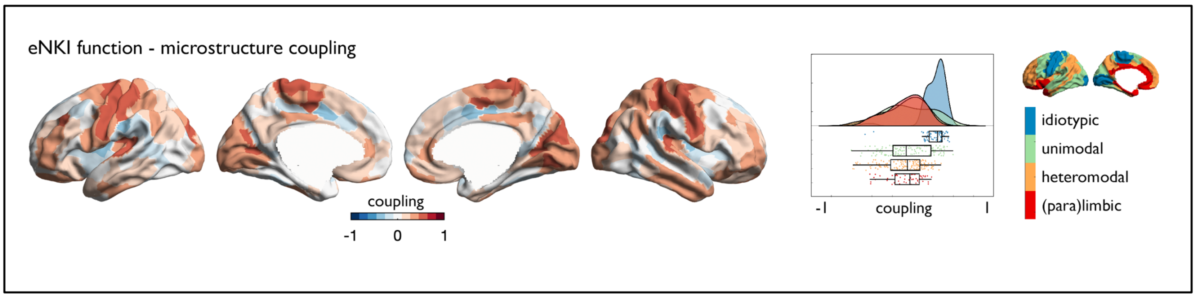

Supplementary Fig 7. Structure-function coupling in dataset processed comparable to the rsFC of the macaque sample. Row-wise coupling projected on the cortical surface and distribution of coupling in 400 parcels across cytoarchitectural classes, boxes show the median and interquartile (25-75%) range, whiskers depict the 1.5*IQR from the quartile. Source data are provided as a Source Data file.

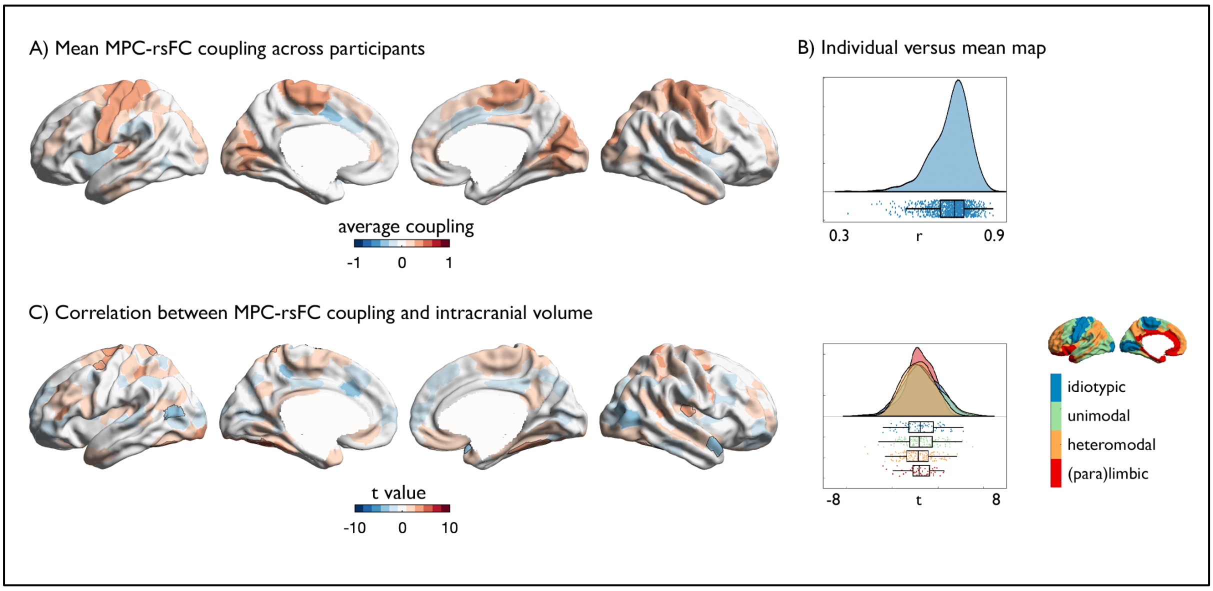

Supplementary Fig 8. Individual variation in structure-function coupling. A) Mean MPC-rsFC coupling across participants; **B)** Association between individual MPC-rsFC maps and mean MPC-rsFC map, and rainbowplot on distribution of correlation of individual coupling maps (n=992) and mean coupling map, boxes show the median and interquartile (25-75%) range, whiskers depict the 1.5*IQR from the quartile; **C)** Correlation between MPC-rsFC coupling and intracranial volume per parcel and distribution of parcels (400) averaged within cytoarchitectural class [6], boxes as in **B**). Source data are provided as a Source Data file.

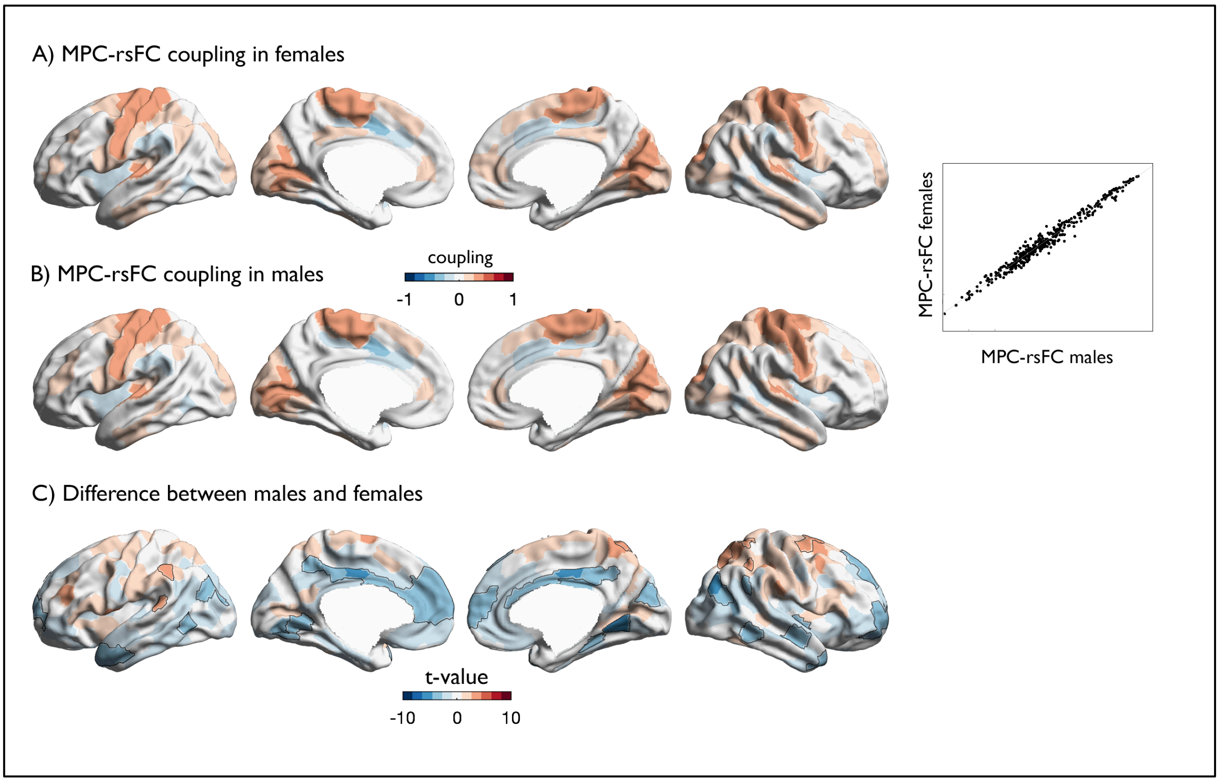

Supplementary Fig 9. Sex differences in structure-function coupling. A) Structure-function coupling in females and **B)** males, and *right* their intercorrelation; **C)** Difference between males and females, red indicates males have stronger coupling than females and blue indicates females have stronger coupling than males. Black outline indicates differences at FDRq<0.05. Source data are provided as a Source Data file.

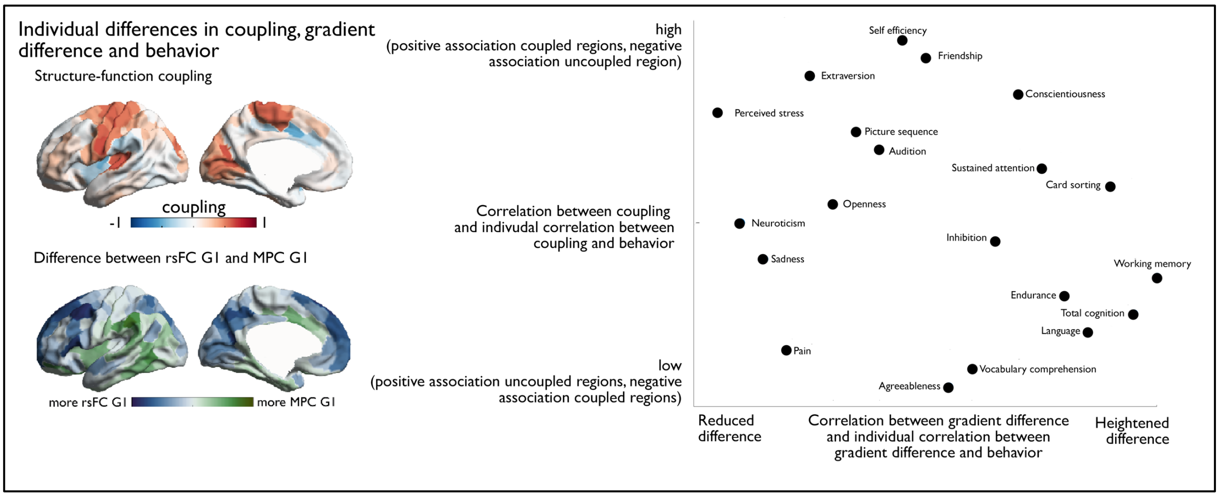

Supplementary Fig 10. Individual level correlations with structure-function coupling along 2D framework. Source data are provided as a Source Data file.

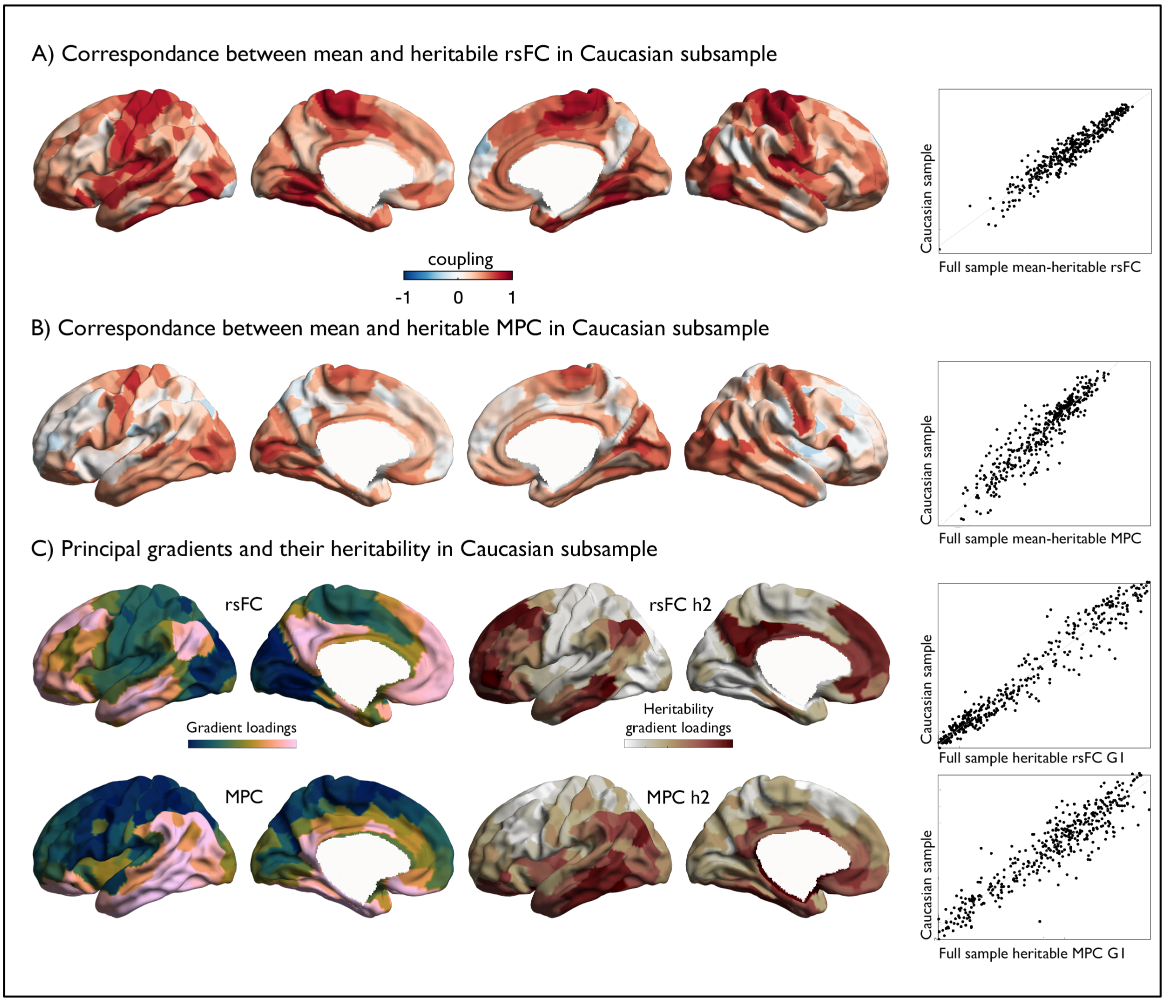

Supplementary Fig 11. Heritability observations in Caucasian subsample. A) Correspondence between mean and heritable rsFC in Caucasian subsample and similarity with full study sample; **B)** Correspondence between mean and heritable MPC in Caucasian subsample and similarity with full study sample; **C)** Gradients of rsFC and MPC in Caucasian subsample and similarity between heritable gradients and those of heritable gradients of full study sample for the respective measure. Source data are provided as a Source Data file.

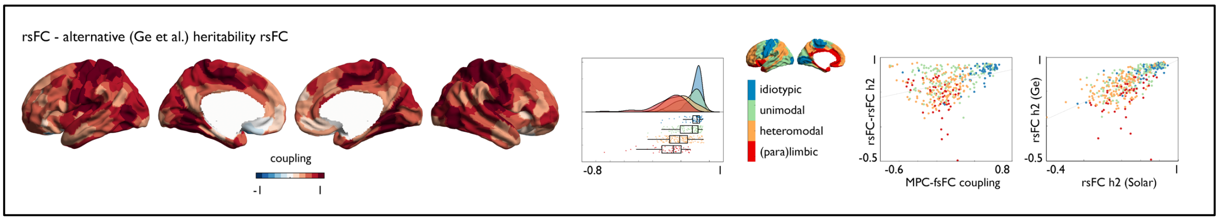

Supplementary Fig 12. rsFC – rsFC heritability coupling considering potential random effects. Scale r_{ranked} [-1 1]; distribution of rsFC-rsFC h2 coupling within cytoarchitectural classes across 400 parcels, boxes show the median and interquartile (25-75%) range, whiskers depict the 1.5*IQR from the quartile; scatter of MPC-rsFC coupling versus rsFC – rsFC h2 coupling (Ge et al.) (colored by cytoarchitectural class); scatter of rsFC-rsFC heritability based on Solar versus rsFC – rsFC h2 coupling based on Ge et al., 2017 (colored by cytoarchitectural class). Source data are provided as a Source Data file.

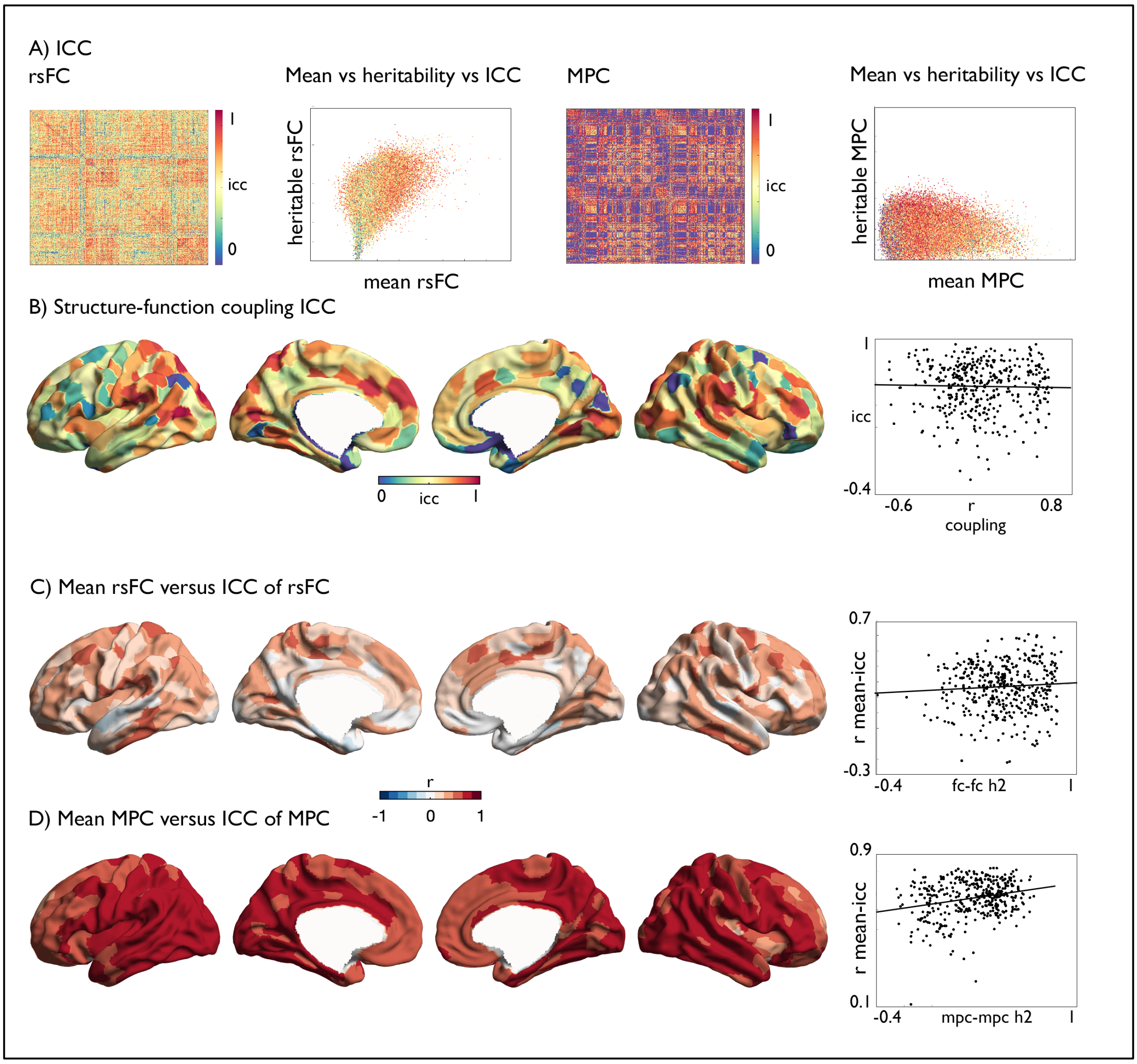

Supplementary Fig 13. Reliability of rsFC, MPC and rsFC-MPC coupling. A) *Left.* ICC of rsFC and association with node-level heritability of rsFC; *right.* ICC of MPC and association with node-level heritability of MPC; **B)** Reliability of structure-function coupling and association of strength of structure-function coupling with reliability of this measure. Colors of ICC indicate poor (ICC<0.5: green and yellow), moderate (0.5<ICC<0.75) purple, and good (ICC>0.75) test-retest reliability; **C**). Seed-wise correlation between mean rsFC and ICC of rsFC; **D**). Seed-wise correlation between mean rsFC and ICC of MPC. Source data are provided as a Source Data file.

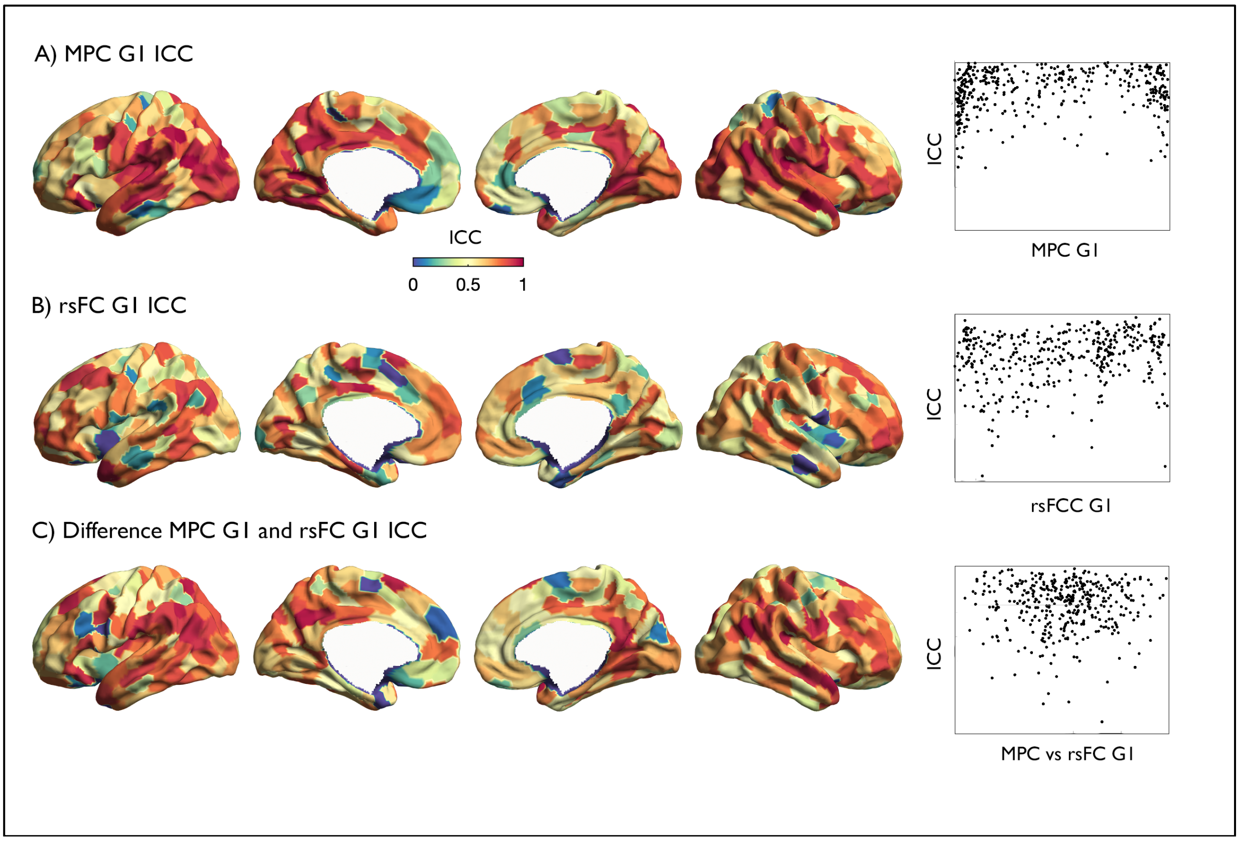

Supplementary Fig 14. Intraclass correlation coefficient (ICC) of MPC and rsFC principal gradients and their difference. A) ICC of MPC-G1; **B**) ICC of rsFC0G1; **C**). ICC of MPC-G1 and rsFC-G1 difference. Source data are provided as a Source Data file.

SUPPLEMENTARY TABLES

Supplementary Table 1. Structure-function coupling mean(SD) as a function of cytoarchitectural class in humans and macaques.

Sample	Idiotypic (mean(SD))	Unimodal	Heteromodal	Paralimbic
**HCP**	0.465(0.181)	0.149(0.263)	0.063(0.198)	0.012(0.146)
**Davis**	0.376(0.260)	0.346(0.221)	0.333(0.255)	0.052(0.230)
**Newcastle**	0.322(0.167)	0.262(0.156)	0.249(0.222)	0.108(0.245)
**Oxford**	0.433(0.255)	0.390(0.224)	0.403(0.269)	0.166(0.284)

Supplementary Table 2. rsFC>MPC. List of genes that showed an increase along the gradient from rsFC to MPC in humans, r>0.5

ACAN	CHGA	FAM162B	HR	MAGI3	PLCB4	SCRT1	TFAM
ACVR1C	CHML	FAM19A2	HSPB8	MAP3K13	PLXDC1	SDSL	THAP10
ADAM23	CITED2	FAM20A	HTR1F	MAP9	PNLDC1	SEMA7A	TIFA
AHI1	CLEC2L	FANCI	IER2	MCF2	POU6F2	SERTAD4	TNNT2
ALDH1A3	CMYA5	FBLN7	IGFBP2	MIR31HG	PPARGC1A	SH3RF2	TPBG
ANK1	CPLX1	FBXO32	INA	MKX	PPL	SHD	TPTE2P6
ANKH	CPNE9	FBXO33	JDP2	MOGAT1	PRDM2	SHROOM2	TRPC3
ANKRD29	CTXN3	FER1L4	KCNA1	MPP1	PRMT7	SIX4	TTC39A
ANKRD34C	DCBLD2	FES	KCNA2	MRPL33	PRRX1	SLC16A6	TTC39B
AR	DCLK1	FGF18	KCNAB3	MYH7B	PRSS16	SLC16A7	UPP1
ARHGAP9	DCUN1D2	FGF9	KCNB1	NAALAD2	PTH2R	SLC24A2	VAMP1
ASB13	DENND2D	FLT3	KCNC1	NCALD	PVALB	SLC25A37	VILL
ATG4D	DEXI	FMN1	KCNC3	NECAB3	QRFPR	SLC38A1	VSTM1
ATRNL1	DMKN	FNDC4	KCNS1	NEFH	RAB37	SLC39A14	VSTM2A
AVPI1	DSCC1	FNDC5	KCNS2	NGB	RAD54B	SOHLH1	ZADH2
BEND6	ECM1	FSTL1	KCTD9	NIPAL2	RAMP3	SORL1	ZBTB8OS
BHLHE40	EEPD1	GABRD	KIAA1107	NR1D2	RARB	SPAG4	ZMAT4
C6orf106	EFNA5	GAS2	KLF9	NR3C1	RCAN2	SPTSSB	ZNF365
CABP1	EFR3A	GLCCI1	KNG1	NT5M	RELL2	SRPK1	ZNF385B
CADPS2	EIF4E1B	GLRX	LAG3	NXPH3	RET	ST8SIA1	
CAMK2G	EIF5A2	GLRX2	LAMA2	ONECUT2	RFPL1	STAC2	
CBLN2	ELMO3	GLS2	LAPTM4B	OSBPL1A	RHBDL3	STAMBPL1	
CCDC39	EPHB6	GPLD1	LCP2	OSBPL3	RHOBTB2	STARD5	
CCDC58	EPN3	GPR161	LINC00473	OSBPL6	RILP	STAT4	
CCNI	ESRRA	GPX3	LINC00515	P2RX6	RORA	STRBP	
CDC42EP3	ESRRG	GSTT1	LRRC38	P2RX6P	RORB	STS	
CDS1	ETV6	GUCA2B	LRRC49	PCDH12	RPP25	SV2C	
CEND1	EXTL2	HAPLN4	LUZP1	PCP4	RTKN2	SYCP2	
CEP152	FAM110A	HIST1H1D	MADCAM1	PCP4L1	SCN1A	SYT2	
CERK	FAM135B	HIVEP2	MAFB	PLCB1	SCN1B	TC2N	

Supplementary Table 3. MPC>rsFC. List of genes that showed an increase along the gradient from MPC to rsFC in humans, r>0.5.

ANKRD50	FKBP1A	LINC00260	PLXNC1	SSTR1
ANKRD6	FNBP1L	LOC100129291	PNMT	SULF2
ASCL2	GABRA5	LOC642852	PON3	SYT17

ATOH7	GABRB1	LOXL1	PPM1M	TEKT2
ATP2B4	GABRE	LRRC36	PPP4R4	THRA
B9D1	GLOD4	LRRC3B	PRKCD	TLL1
BIRC3	GMFB	LUZP2	PRKCG	TMEFF2
CADM1	GNG10	LXN	PSD3	TMEM159
CAMK2D	GNG2	MACROD2	PTCHD1	TMEM200A
CD24	GNG4	MAPK1	PTGER3	TNFRSF11A
CFD	GPC4	MARCKS	PTPRA	TUBB2A
CLEC4G	GPD2	MESP1	PTPRR	UCHL3
COCH	GPR160	MMD	PYDC1	WDR66
CPE	GRIK2	NANOS1	RAB27B	WDR86
CPNE6	GRP	NCAM2	RAPGEF4	XKR4
CPNE7	HPCAL4	NECAB2	RAVER2	YPEL1
CTXN1	HSPB2	NKAIN4	RILPL2	ZCCHC12
CXorf57	HTR1A	NOV	RNF150	ZCCHC17
DACH2	HTR2C	NPPA	RRP7A	ZCCHC18
DHDH	IL13RA2	NR2F2	RSPH9	ZNF436
DIAPH2	IQCJ	NTSR1	SCGN	
DIRAS3	ISG15	NUDT11	SCN3B	
DOK6	ISOC1	OPRM1	SCN9A	
DPYSL3	KCNA4	PCSK5	SHF	
DYDC2	KCNG1	PDYN	SLA	
EFCAB1	KCNN3	PEA15	SLC16A2	
F12	KCTD12	PGAP1	SLC17A8	
FABP7	KCTD4	PGRMC1	SLIT1	
FAM171B	KLHL13	PID1	SLIT3	
FAM71F1	KLK10	PKIA	SMARCD3	

Supplementary Table 4. Coupling List of genes that showed an increase with increased coupling in humans, r>0.5.

ACAN	CCNI	EIF5A2	GPLD1	KNG1	NECAB3	RAB37	SIX4	SYT6
ACVR1C	CDC42EP3	ELMO3	GPR161	L2HGDH	NEFH	RAD54B	SLC16A6	TC2N
ACYP2	CDH7	ENTPD4	GSTT1	LAG3	NEFL	RAMP3	SLC16A7	THAP10
ADAM23	CDR2L	EPHB6	GUCA2B	LAPTM4B	NT5M	RBMS1	SLC17A6	THEMIS
ADM	CDS1	EPN3	HAPLN4	LCP2	OIP5-AS1	RCAN2	SLC24A2	TIFA
AHI1	CERK	ESRRA	HIVEP2	LEPROTL1	ONECUT2	RELL2	SLC25A12	TPBG
ALDH1A3	CHAF1A	ESRRG	HR	LINC00515	OR2L3	RELT	SLC25A25	TPTE2P6
ANK1	CHGA	ETV6	HS3ST1	LRRC38	OSBPL1A	RET	SLC25A37	TRAK2
ANKH	CHML	EXTL2	HS3ST5	LRRC49	OSBPL6	RFPL1	SLC25A5	TRMT61B
ANKRD29	CITED2	FAM110A	HSPA4L	LUZP1	OXNAD1	RHBDL3	SLC38A1	TRPC3
ANKRD34C	CLEC2L	FAM135B	HTR1F	LYPLA1	P2RX6	RHOBTB2	SLC39A13	TTC39B
ANKRD42	CMYA5	FAM20A	IER2	LYSMD4	P2RX6P	RILP	SLC39A14	TTC39C
AR	COX7A1	FAM49B	IFFO1	MADCAM1	PCDH12	RIMKLA	SLC4A8	ULK3
ARHGAP9	CPLX1	FAM57B	IGFBP2	MAFB	PCDH7	RORA	SOHLH1	UPP1
ASB13	CPNE9	FAM71E1	INA	MAGI3	PCP4	RORB	SORL1	VAMP1

ATG4D	CRTAC1	FBLN7	IPW	MAP3K13	PCP4L1	RPGR	SPAG4	VAV3
ATRNL1	DCBLD2	FBXO32	ISCU	MAP9	PHYH	RRM2B	SPTSSB	VILL
AVPI1	DCLK1	FBXO33	ITGA11	MAT2B	PLCB1	RSPO2	SRPK1	VSTM1
BCAT1	DCUN1D2	FER1L4	ITM2A	MCF2	PLCB4	RTKN2	ST3GAL6	VSTM2A
BEND6	DDHD2	FES	JDP2	MGST2	PLXDC1	SAP30L	ST8SIA1	ZADH2
BHLHE40	DEXI	FGF18	KCNA1	MIR31HG	POSTN	SCN1A	STAC2	ZBTB8OS
BLMH	DMKN	FGF9	KCNA2	MKX	POU6F2	SCN1B	STAMBPL1	ZMAT4
C17orf75	DNAJC4	FMN1	KCNAB3	MOGAT1	PPARGC1A	SCN4B	STARD10	ZNF365
C3orf18	ECM1	FNDC5	KCNB1	MPP1	PPIL3	SCRT1	STARD5	
C6orf106	ECSIT	FRAT1	KCNC1	MRPL33	PPTC7	SDSL	STAT4	
CABP1	EEPD1	FSTL1	KCNC3	MX1	PRMT7	SEMA7A	STRBP	
CACNA2D2	EFNA5	GAS2	KCNS1	MYH7B	PRSS16	SERTAD4	STS	
CAMK2G	EFR3A	GLCCI1	KCNS2	NAALAD2	PVALB	SH3BGRL	SV2C	
CCDC39	EIF4A2	GLRX2	KCTD9	NCOA3	PXDNL	SHD	SYCP2	
CCDC58	EIF4E1B	GNA14	KLF9	NEB	QRFPR	SHROOM2	SYT2	

Supplementary Table 5. Uncoupling List of genes that showed a decrease with increased coupling in humans, r>0.5.

ADAMTS3	GABRA3	NOL4	SLC17A8
AMIGO2	GABRB1	NPY1R	SLIT1
ANKRD50	GABRE	NR2F2	SLIT3
ANKRD6	GLOD4	NTSR1	SLN
ARHGAP4	GNB4	NUDT11	SYN2
ASCL2	GNG10	ONECUT1	TEKT2
ATOH7	GNG2	OPRM1	THRA
ATP2B4	GNG4	PCSK5	TLL1
B9D1	GPD2	PGAP1	TMEFF2
CAMK2D	GPR160	PGRMC1	TMEM159
CCBE1	GRIK2	PKIA	TMEM200A
CCDC85C	HMGCS1	PLXNC1	TNFRSF11A
CD24	HPCAL4	PNMT	TUBB2A
CFD	HTR2C	PPM1M	UCHL3
CHMP1A	IL13RA2	PPP4R4	WDR66
CNR1	IQCJ	PRKCD	XKR4
CPNE6	KCNG1	PSD3	YPEL1
DHDH	KLK10	PTCHD1	ZCCHC12
DNAH14	KLK7	PTGER3	ZCCHC17
DNAJA4	LINC00260	PTPRR	ZCCHC18
DPYSL3	LOC100129291	PYDC1	ZNF436
DYDC2	LRRC36	RAPGEF4	
EFCAB1	LRRC3B	RAVER2	
FABP7	MAPK1	RNF150	
FAM110C	MARCKS	RRP7A	
FAM171B	MESP1	RSPH9	

FAM71F1	MYL5	SCGN
FKBP1A	NANOS1	SCN9A
FNBP1L	NCAM2	SHF
FXYD6	NKAIN4	SLA

Supplementary References

1 Yeo, B. T. *et al.* The organization of the human cerebral cortex estimated by intrinsic functional connectivity. *J Neurophysiol* **106**, 1125-1165, doi:10.1152/jn.00338.2011 (2011).

2 Paquola, C. *et al.* Shifts in myeloarchitecture characterise adolescent development of cortical gradients. *Elife* **8**, doi:10.7554/eLife.50482 (2019).

3 Markov, N. T. *et al.* A weighted and directed interareal connectivity matrix for macaque cerebral cortex. *Cereb Cortex* **24**, 17-36, doi:10.1093/cercor/bhs270 (2014).

4 Xu, T. et al. Cross-species functional alignment reveals evolutionary hierarchy within the connectome. Neuroimage **223**, 117346, doi:10.1016/j.neuroimage.2020.117346 (2020).

5 Ge, T., Holmes, A. J., Buckner, R. L., Smoller, J. W. & Sabuncu, M. R. Heritability analysis with repeat measurements and its application to resting-state functional connectivity. *Proc Natl Acad Sci U S A* **114**, 5521-5526, doi:10.1073/pnas.1700765114 (2017).

6 Mesulam M-M. Behavioral neuroanatomy: Largescale networks, association cortex, frontal syndromes, the limbic system, and hemispheric specialization. In: Principles of Behavioral and Cognitive Neurology. p. 1–120. (Oxford press, 2000)

7 Margulies, D. S. *et al.* Situating the default-mode network along a principal gradient of macroscale cortical organization. *Proc Natl Acad Sci U S A* **113**, 12574-12579, doi:10.1073/pnas.1608282113 (2016).

8 Paquola, C. *et al.* Microstructural and functional gradients are increasingly dissociated in transmodal cortices. *PLoS Biol* **17**, e3000284, doi:10.1371/journal.pbio.3000284 (2019).
