# Supplementary material for: Genetic and phylogenetic uncoupling of structure and function in human transmodal cortex
Source: Nat Commun. 2022 May 9;13:2341. doi: 10.1038/s41467-022-29886-1 (PMC9085871; doi:10.1038/s41467-022-29886-1)
Supplement: Supplementary file 2 — Description of Additional Supplementary Information [file 41467_2022_29886_MOESM2_ESM.docx]

**Description of Additional Supplementary Files**

File Name: Supplementary Materials.

Description: Supplementary results, figures and tables.

- Replication and robustness analysis (Supplementary Fig 2 and 3)
- Heritability and variance of principle gradient of MPC and rsFC (Fig 5)
- Macaque gradient validation (Supplementary Fig 4)
- Robustness evaluations of human- macaque differences
- Individual variation in humans
- Alternative heritability computations considering intra-individual variation (Supplementary Fig 12)
- Reliability using test-retest analysis
