# Supplementary material for: Genetic and phylogenetic uncoupling of structure and function in human transmodal cortex
Source: Nat Commun. 2022 May 9;13:2341. doi: 10.1038/s41467-022-29886-1 (PMC9085871; doi:10.1038/s41467-022-29886-1)
Supplement: Supplementary file 4 — Reporting Summary [file 41467_2022_29886_MOESM4_ESM.pdf]

nature portfolio

Corresponding author(s): Sofie Louise Valk

Last updated by author(s): Feb 16, 2022

Reporting Summary

Nature Portfolio wishes to improve the reproducibility of the work that we publish. This form provides structure for consistency and transparency in reporting. For further information on Nature Portfolio policies, see our Editorial Policies and the Editorial Policy Checklist.

Statistics

For all statistical analyses, confirm that the following items are present in the figure legend, table legend, main text, or Methods section.

n/a	Confirmed	
☐	☒	The exact sample size (*n*) for each experimental group/condition, given as a discrete number and unit of measurement
☐	☒	A statement on whether measurements were taken from distinct samples or whether the same sample was measured repeatedly
☐	☒	The statistical test(s) used AND whether they are one- or two-sided<br>*Only common tests should be described solely by name; describe more complex techniques in the Methods section.*
☐	☒	A description of all covariates tested
☐	☒	A description of any assumptions or corrections, such as tests of normality and adjustment for multiple comparisons
☐	☒	A full description of the statistical parameters including central tendency (e.g. means) or other basic estimates (e.g. regression coefficient) AND variation (e.g. standard deviation) or associated estimates of uncertainty (e.g. confidence intervals)
☐	☒	For null hypothesis testing, the test statistic (e.g. F, t, r) with confidence intervals, effect sizes, degrees of freedom and P value noted<br>*Give P values as exact values whenever suitable.*
☒	☐	For Bayesian analysis, information on the choice of priors and Markov chain Monte Carlo settings
☐	☒	For hierarchical and complex designs, identification of the appropriate level for tests and full reporting of outcomes
☒	☐	Estimates of effect sizes (e.g. Cohen's *d*, Pearson's *r*), indicating how they were calculated

Our web collection on statistics for biologists contains articles on many of the points above.

Software and code

Policy information about availability of computer code

Data collection	Human data analyzed in our main results were obtained from the open-access HCP S1200 young adult sample (HCP; http://www.humanconnectome.org/). Macaque data was obtained from PRIME-DE (http://fcon_1000.projects.nitrc.org/indi/indiPRIME.html; University of California, Davis). Supplementary analysis were performed using the MICS dataset (https://portal.conp.ca/dataset?id=projects/mica-mics) and eNKI dataset (http://fcon_1000.projects.nitrc.org/indi/enhanced/).
Data analysis	Both resting-state fMRI and T1wT2w data were derived from the HCP minimal processing pipeline (http://www.humanconnectome.org/) and can be acquired through https://db.humanconnectome.org/ with the acceptance of HCP Open Access Data Use Terms.<br>MPC was constructed using (https://github.com/MICA-MNI/MPC, see also https://github.com/MICA-MNI/micapipe), using Freesurfer 6.0<br>Heritability analyses were performed using Solar Eclipse 8.4.0 (http://www.solar-eclipse-genetics.org), and data on the pedigree analysis is available here: https://www.nitrc.org/projects/se_linux/<br>To preprocess T1wT2w macaque data we used a customized HCP-like pipeline (doi:10.5281/record/zenodo.3888969). The macaque monkey intrinsic functional data were preprocessed using a customized Connectome Computational System (https://github.com/zuoxinian/CCS). These scripts were ran using Freesurfer 5.3; FSL 6.0; ANTs 2.1.0; workbench 1.4.2; MSM 1.0.0; AFNI 18.0.21.<br>Alignment between human and macaque surfaces was performed using joint embedding (https://github.com/TingsterX/PRIME-DE)<br>Connectome manifold generation using BrainSpace (https://github.com/MICA-MNI/BrainSpace), using Matlab 2020a.<br>Transcriptomic association analyses using NeuroVault (https://neurovault.org/), cell-type specific expression analysis (CSEA) tool (http://genetics.wustl.edu/jdlab/csea-tool-2), and abagen (https://github.com/rmarkello/abagen).<br>Functional decoding was performed using Neurosynth (https://github.com/neurosynth/neurosynth)<br>Further custom code is provided here: https://github.com/CNG-LAB/cngopen/tree/main/structure_function and Zenodo doi:10.5281/zenodo.6141483

For manuscripts utilizing custom algorithms or software that are central to the research but not yet described in published literature, software must be made available to editors and reviewers. We strongly encourage code deposition in a community repository (e.g. GitHub). See the Nature Portfolio guidelines for submitting code & software for further information.

Data

Policy information about availability of data

All manuscripts must include a data availability statement. This statement should provide the following information, where applicable:

- Accession codes, unique identifiers, or web links for publicly available datasets
- A description of any restrictions on data availability
- For clinical datasets or third party data, please ensure that the statement adheres to our policy

This study followed institutional review board guidelines of corresponding institutions. Human data analyzed in our main results were obtained from the open-access HCP S1200 young adult sample (HCP; http://www.humanconnectome.org/). Macaque data was obtained from PRIME-DE (http://fcon_1000.projects.nitrc.org/indi/indiPRIME.html; University of California, Davis). Heritability analyses were performed using Solar Eclipse 8.4.0 (http://www.solar-eclipse-genetics.org), and data on the pedigree analysis is available here: https://www.nitrc.org/projects/se_linux/ 106,133. Gradient mapping analyses was based on BrainSpace (https://brainspace.readthedocs.io/en/latest/). Transcriptomic association analyses were conducted using NeuroVault (https://neurovault.org), abagen tools (https://github.com/rmarkello/abagen)94, and cell-type-specific expression analysis (CSEA) (http://genetics.wustl.edu/jdlab/csea-tool-2)120 Supplementary analysis were performed using the MICS dataset (https://portal.conp.ca/dataset?id=projects/mica-mics) and eNKI dataset (http://fcon_1000.projects.nitrc.org/indi/enhanced/). Source data are provided with this paper and code for visualization of parcel results on surface linked in our study's Github repository (https://github.com/CNG-LAB/cngopen/tree/main/structure_function).

Field-specific reporting

Please select the one below that is the best fit for your research. If you are not sure, read the appropriate sections before making your selection.

☒ Life sciences ☐ Behavioural & social sciences ☐ Ecological, evolutionary & environmental sciences

For a reference copy of the document with all sections, see nature.com/documents/nr-reporting-summary-flat.pdf

Life sciences study design

All studies must disclose on these points even when the disclosure is negative.

Sample size	Our main human sample (HCPS1200) consisted of 992 (529 females) individuals (including 255 MZ-twins and 150 DZ-twins) with an age mean ±SD=28.71±3.72 years (range =22-37 years). The main macaque dataset (UC Davis) consisted of 19 rhesus macaque monkeys (macaca mulatta, all female, age±SD=20.38±0.93 years, weight=9.70±1.58 kg).
Data exclusions	We removed individuals with missing structural and functional imaging data (e.g. not 4 complete sessions) (HCP). We did not exclude data from the macaque dataset.
Replication	Human data:<br>MICs dataset: 50 healthy volunteers (21 women; age mean±SD=29.82±5.73 years; 47 right-handed).<br>eNKI dataset: N=100, age-range 18-40yrs.<br>Test-retest sample of the HCPS1200 release.<br>Macaque:<br>Newcastle dataset (awake) consisted of 10 rhesus macaques (8 males, age mean±SD=8.28±2.33, weight=11.76±3.38).<br>Oxford dataset (anesthetized), we included nineteen rhesus macaques with preprocessing and surface reconstruction (all males, age=4.01 ±0.98 years, weight=6.61±2.04 kg).<br>All replications yielded broadly similar patterns of structure-function coupling, gradient organization, and cross-species differences.
Randomization	For heritability analysis we controlled for effects of age, sex, age2, and age times sex. Main findings of structure-function coupling and organization were performed on mean of the sample. Follow-up analysis controlling for covariates (age, sex, intracranial volume) indicated effects of structure-function coupling were consistent at the level of the individual.
Blinding	Blinding was not relevant to this study. We need to know the participants' relateness information to select them for heritability analysis.

Reporting for specific materials, systems and methods

We require information from authors about some types of materials, experimental systems and methods used in many studies. Here, indicate whether each material, system or method listed is relevant to your study. If you are not sure if a list item applies to your research, read the appropriate section before selecting a response.

Materials & experimental systems

n/a	Involved in the study
☒	☐ Antibodies
☒	☐ Eukaryotic cell lines
☒	☐ Palaeontology and archaeology
☐	☒ Animals and other organisms
☐	☒ Human research participants
☒	☐ Clinical data
☒	☐ Dual use research of concern

Methods

n/a	Involved in the study
☒	☐ ChIP-seq
☒	☐ Flow cytometry
☐	☒ MRI-based neuroimaging

Animals and other organisms

Policy information about studies involving animals; ARRIVE guidelines recommended for reporting animal research

Laboratory animals	The current study used open data.
Wild animals	N/A
Field-collected samples	We used open data from the the recently established PRIME-DE (http://fcon_1000.projects.nitrc.org/indi/indiPRIME.html). Davis: The neuroimaging experiments and associated procedures were performed at the California National Primate Research Center (CNPRC) under protocols approved by the University of California, Davis Institutional Animal Care and Use Committee (see further Baxter, 2018). For replication we used: Newcastle dataset (Rinne, 2017; Schonwiesner, 2015) and Oxford dataset (Noonan, 2014). Both datasets received ethics from their local institution.
Ethics oversight	The HHU approved of using the open datasets and analysis in the context of the current study.

Note that full information on the approval of the study protocol must also be provided in the manuscript.

Human research participants

Policy information about studies involving human research participants

Population characteristics	HCP: We included individuals for whom the scans and data had been released after passing the HCP quality control and assurance standards. The full set of inclusion and exclusion criteria are described elsewhere. In short, the primary participant pool comes from healthy individuals born in Missouri to families that include twins, based on data from the Missouri Department of Health and Senior Services Bureau of Vital Records. Additional recruiting efforts were used to ensure participants broadly reflect ethnic and racial composition of the U.S. population. Healthy is broadly defined, in order to gain a sample generally representative of the population at large. Sibships with individuals having severe neurodevelopmental disorders (e.g., autism), documented neuropsychiatric disorders (e.g., schizophrenia or depression) or neurologic disorders (e.g., Parkinson's disease) are excluded, as well as individuals with diabetes or hypertension. Twins born prior to 34 weeks of gestation and non-twins born prior to 37 weeks of gestation were excluded. After removing individuals with missing structural and functional imaging data, our sample consisted of 992 (529 females) individuals (including 255 MZ-twins and 150 DZ-twins) with an age mean±SD=28.71±3.72 years (range =22-37 years).<br>MICS: Data were collected in a sample of 50 healthy volunteers (21 women; age mean±SD=29.82±5.73 years; 47 right-handed) between April 2018 and September 2020 Royer, biorXiv, Each participant underwent a single testing session. All participants denied a history of neurological and psychiatric illness. The Ethics Committee of the Montreal Neurological Institute and Hospital approved the study. Written informed consent, including a statement for openly sharing all data in anonymized form, was obtained from all participants<br>eNKI: To evaluate whether differences in rs-fMRI pre-processing between humans and macaques can explain differences observed in coupling of MPC and rsFC between both species, we evaluated structure-function coupling in an additional human sample (eNKI subsample. N=100, age-range 18-40yrs) preprocessed analogously to the macaque datasets, including temporal compression, motion correction, 4D global scaling, nuisance regression (Friston's 24 model, cerebrospinal fluid and white matter), linear and quadratic detrends, band-pass filtering (0.01–0.1 Hz), and surface registration (for details, see Nenning, 2020). For more information see: http://fcon_1000.projects.nitrc.org/indi/enhanced/recruit.html
Recruitment	Please see above.
Ethics oversight	The current research complies with all relevant ethical regulations as set by The Independent Research Ethics Committee at the Medical Faculty of the Heinrich-Heine-University of Duesseldorf (study number 2018-317).

Note that full information on the approval of the study protocol must also be provided in the manuscript.

Magnetic resonance imaging

Experimental design

Design type	Resting-state and anatomical study

Design specifications

N/A

Behavioral performance measures

For supplementary analysis we evaluated the association between structure-function coupling and gradient difference and individual-level behavior, we selected 20 markers of individual difference from the HCP battery i.e., total cognition, card sorting, list sorting, friendship, picture vocabulary, reading English, pain, endurance, flanker, picture sequence, self-efficiency, perceived stress, noise, SCPT, sadness, and NEO-FFI 50.

Acquisition

Imaging type(s)

In short, MRI data used in the study were acquired on the HCP's custom 3T Siemens Skyra equipped with a 32-channel head coil. Two T1w images with identical parameters were acquired using a 3D-MPRAGE sequence (0.7 mm isovoxels, matrix=320×320, 256 sagittal slices; TR=2,400 ms, TE=2.14 ms, TI=1,000 ms, flip angle=8°; iPAT=2). Two T2w images were acquired using a 3D T2-SPACE sequence with identical geometry (TR=3,200 ms, TE=565 ms, variable flip angle; iPAT=2). T1w and T2w scans were acquired on the same day.

Supplementary samples:
MICS:Two T1w scans with identical parameters were acquired with a 3D magnetization-prepared rapid gradient-echo sequence (MP-RAGE; 0.8mm isovoxels, matrix=320×320, 224 sagittal slices, TR=2,300ms, TE=3.14ms, TI=900ms, flip angle=9°, iPAT=2, partial Fourier=6/8). Both T1w scans were visually inspected to ensure minimal head motion before they were submitted to further processing. qT1 relaxometry data were acquired using a 3D-MP2RAGE sequence (0.8mm isovoxels, 240 sagittal slices, TR=5000ms, TE=2.9ms, TI 1=940ms, T1 2=2830ms, flip angle 1=4°, flip angle 2=5°, iPAT=3, bandwidth=270 Hz/px, echo spacing=7.2ms, partial Fourier=6/8). We combined two inversion images for qT1 mapping in order to minimize sensitivity to B1 inhomogeneities and optimize intra- and intersubject reliability 121,122. One 7 min rs-fMRI scan was acquired using multiband accelerated 2D-BOLD echo-planar imaging (3mm isotropic voxels, TR=600ms, TE=30ms, flip angle=52°, FOV=240×240mm2, slice thickness=3mm, mb factor=6, echo spacing=0.54ms). Participants were instructed to keep their eyes open, look at a fixation cross, and not fall asleep. We also include two spin-echo images with reverse phase encoding for distortion correction of the rs-fMRI scans (3mm isotropic voxels, TR=4029ms, TE=48ms, flip angle=90°, FOV=240×240mm2, slice thickness=3mm, echo spacing=0.54ms, phase encoding=AP/PA, bandwidth= 2084 Hz/Px).
eNKI: For each individual, a high-resolution T1-weighted scan (1 mm isotropic resolution, TR = 1900 ms, TE = 2.52 ms, Flip angle = 9°) and a 10-min rs-fMRI acquisition (3 mm isotropic resolution, TR = 645 ms, TE = 30 ms, Flip angle = 60°) were included in the current study. The details of the dataset and sequences are described elsewhere (
Macaque main sample: The resting-state fMRI data were collected with 1.4×1.4×1.4 mm resolution, TR=1.6s, 6.67 min (250 volumes) under anesthesia. No contrast-agent was used during the scans. Structural data (T1w and T2w) were acquired with 0.3×0.3×0.3 mm resolution (T1w: TR=2,500ms, TE=3.65ms, TI=1,100ms, flip angle=7 degrees, FOV=154mm; T2w: TR=3,000ms, TE=307ms). See further: http://fcon_1000.projects.nitrc.org/indi/enhanced/
Supplementary macaque samples:
Newcastle: The fMRI session was acquired with 1.2×1.2×1.2 mm resolution, TR=2,000 ms, 8.33-min per scan (250 volumes x 2 scan) per animal.
Oxford: Resting-state fMRI (rs-fMRI) data were collected with 2 mm isotropic resolution, TR=2,000 ms, 53.3 min (1,600 volumes). No contrast-agent was used during the scans.

Field strength

3T

Sequence & imaging parameters

please see above

Area of acquisition

whole brain field of view

Diffusion MRI ☐ Used ☒ Not used

Preprocessing

Preprocessing software

For HCP data we used the standard preprocessed data, including co-registration of T1- and T2-weighted scans, B1 (bias field) correction, and segmentation and surface reconstruction using FreeSurfer version 5.3-HCP. Using these data, the equidistant surfaces are computed for MPC measurement. Functional connectivity matrices were based on 1-hour of resting-state fMRI data acquired through the HCP, which underwent HCP's minimal preprocessing. Briefly, for each individual, a functional connectivity matrix was calculated using the correlation coefficient of the average of the four minimally preprocessed, spatially normalized, and concatenated to 4 15-min resting-state fMRI scans which were co-registered using MSMAll to template HCP 32k_LR surface space 50. 32k_LR surface space consists of 32,492 total nodes per hemisphere (59,412 excluding the medial wall). Following average time-series were extracted in each of the 400 cortical parcels 58 and individual functional connectivity matrices were computed. The individual functional connectomes were generated by averaging preprocessed timeseries within nodes, correlating nodal timeseries and converting them to z scores. Here we used the individual timeseries of individuals with complete data in the S1200 sample.

For the macaque data data we used a modified version of the human connectome project preprocessing pipeline, using FSL, Freesurfer, and ANTs normalization, and ITK-snap for manual corrections (see further https://zenodo.org/record/3888969).

MICs: Raw DICOMS were sorted by sequence into distinct directories using custom scripts. Sorted files were converted to NIfTI format using dcm2niix (v1.0.20200427; https://github.com/rordenlab/dcm2niix) renamed, and assigned to their respective subject-specific directories according to BIDS standards.As previously described rs-fMRI data were pre-processed using a combination of AFNI and FSL. To ensure magnetic field saturation, the first five volumes were disregarded. Images were reoriented, motion and distortion corrected. Motion correction was performed by registering all timepoints to the mean volume, while distortion correction leveraged main phase and reverse phase field maps acquired alongside rs-fMRI

scans. Nuisance variable signal was removed using an in-house trained ICA-FIX classifier and via spike regression using motion outlier outputs provided by FSL. Volumetric timeseries were averaged for registration to native FreeSurfer space using boundary-based registration, and mapped to individual surface models using trilinear interpolation. Native surface cortical timeseries underwent spatial smoothing once mapped to each individual's cortical surface models (Gaussian kernel, FWHM=10mm), and were subsequently averaged within nodes defined by the Schaefer 400 parcellation.

eNKI: (based on Nenning,2020) The structural MRI preprocessing: spatial denoising (CAT12), brain extraction, segmentation, and surface reconstruction. The deformation sphere from the native surface to fsaverage space was calculated in FreeSurfer. Nenning et al. generated the standard surface meshes, for which each node has one-to-one correspondence with a node on the high-resolution fsaverage surface in native space. The functional image preprocessing included discarding the first five timepoints, compressing temporal spikes (AFNI 3dDespike), slice timing correction, motion correction, 4D global mean intensity normalization, nuisance regression (Friston's 24 model, cerebrospinal fluid and white matter), linear and quadratic detrends, as well as band-pass filtering (0.01–0.1 Hz). The preprocessed data were then registered to the anatomical space using boundary-based registration and projected to the high-resolution surface (i.e. fsaverage) in native space. The rs-fMRI data were then spatially smoothed on the fsaverage surface (FWHM = 6 mm). The data were downsampled to fsaverage4 surface for the following analyses.

Normalization: please see above.

Normalization template: HCP: T1wT2w: individual participant space, and averaged across Schaefer parcel (replicated in Glasser atlas). rsfMRI: HCP 32k_LR surface space; macaque data: Yerkes19 macaque surface template; MICs data: individual templates and Schaefer parcels; eNKI data: fsaverage4

Noise and artifact removal: please see above.

Volume censoring: please see above.

Statistical modeling & inference

Model type and settings: Main analysis include univariate correlations between structure-function associations and we modeled organization of MPC and rsFC using non-linear dimensionality reduction techniques (diffusion map embedding, brainspace.readthedocs.io)

Effect(s) tested: We evaluated heritability and structure-function coupling.

Specify type of analysis: ☐ Whole brain ☒ ROI-based ☐ Both

Anatomical location(s): We used the Schaefer parcellation (400 regions) and the Glasser parcellation (360 regions) to average our structural and functional data. In case of the macaque data, we used the Markov parcellation (182 regions).

Statistic type for inference (See Eklund et al. 2016): We did not use voxel-wise or cluster-wise statistics.

Correction: Spin tests for spatial autocorrelation and false discovery rate correction to adjust for multiple comparisons.

Models & analysis

n/a	Involved in the study
☐	☒ Functional and/or effective connectivity
☒	☐ Graph analysis
☒	☐ Multivariate modeling or predictive analysis

Functional and/or effective connectivity: Pearson correlation and Fisher's z transform.
